# From Images to Genes: Radiogenomics Based on Artificial Intelligence to Achieve Non‐Invasive Precision Medicine in Cancer Patients

**DOI:** 10.1002/advs.202408069

**Published:** 2024-11-13

**Authors:** Yusheng Guo, Tianxiang Li, Bingxin Gong, Yan Hu, Sichen Wang, Lian Yang, Chuansheng Zheng

**Affiliations:** ^1^ Department of Radiology Union Hospital Tongji Medical College Huazhong University of Science and Technology Wuhan 430022 China; ^2^ Hubei Key Laboratory of Molecular Imaging Wuhan 430022 China; ^3^ Department of Ultrasound State Key Laboratory of Complex Severe and Rare Diseases Peking Union Medical College Hospital Chinese Academy of Medical. Sciences Peking Union Medical College Beijing 100730 China; ^4^ Research Institute of Trustworthy Autonomous Systems and Department of Computer Science and Engineering Southern University of Science and Technology Shenzhen 518055 China; ^5^ School of Life Science and Technology Computational Biology Research Center Harbin Institute of Technology Harbin 150001 China

**Keywords:** artificial intelligence, cancer, immune microenvironment, medical imaging, multi‐omics analysis, precision medicine, radiomics, single‐cell sequencing

## Abstract

With the increasing demand for precision medicine in cancer patients, radiogenomics emerges as a promising frontier. Radiogenomics is originally defined as a methodology for associating gene expression information from high‐throughput technologies with imaging phenotypes. However, with advancements in medical imaging, high‐throughput omics technologies, and artificial intelligence, both the concept and application of radiogenomics have significantly broadened. In this review, the history of radiogenomics is enumerated, related omics technologies, the five basic workflows and their applications across tumors, the role of AI in radiogenomics, the opportunities and challenges from tumor heterogeneity, and the applications of radiogenomics in tumor immune microenvironment. The application of radiogenomics in positron emission tomography and the role of radiogenomics in multi‐omics studies is also discussed. Finally, the challenges faced by clinical transformation, along with future trends in this field is discussed.

## Graphical abstract

Radiogenomics is an emerging interdisciplinary field with promising prospects in oncology. This review is dedicated to summarizing the current workflows, emphasizing the application of artificial intelligence within them, and engaging in discussions around crucial topics such as tumor heterogeneity and the immune microenvironment in the context of radiogenomics. Finally, it elaborates on the challenges faced by radiogenomics and future trends.

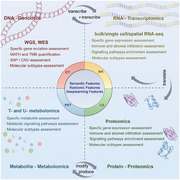

## 1 Background

With the development of high‐throughput sequencing technology, the concept of genomics has been greatly expanded. Initially, genomics was used to determine DNA sequences,^[^ ^1^ ^]^ but it has now expanded into functional genomics such as transcriptomics and proteomics^[^ ^2^ ^]^ and epigenomics such as DNA methylation or RNA methylation.^[^ ^3^ ^]^ Based on the results of genomic sequencing, we can study the deep‐seated biological phenotypes of tumors and further combine them with specific treatment methods to achieve precision medicine.^[^ ^4^, ^5^ ^]^ However, genomics often relies on tumor samples obtained by invasive means such as needle biopsy or surgical resection, which has the following practical problems: patients are intolerant to surgery; the tumor is located next to large blood vessels or vital organs; bleeding, pneumothorax, infection and, other life‐threatening complications occur during puncture or operation; obtaining multiple samples during the course of treatment remains challenging; creating an additional economic burden; the tumor itself exhibit spatial heterogeneity, meaning that the punctured or resected samples may not represent the overall characteristics of the tumor.^[^ ^6^, ^7^, ^8^ ^]^ These problems limit the development and clinical application of genomics.

In the last decade, radiomics based on clinical imaging including computed tomography (CT), magnetic resonance imaging (MRI), positron emission tomography (PET), and ultrasound has been extensively studied and attracted the attention of researchers worldwide.^[^ ^9^ ^]^ Radiomics aims to extract quantitative features from clinical imaging and provide a wealth of relevant information about disease phenotypes that are difficult to distinguish with the naked eye.^[^ ^10^ ^]^ The analysis process of radiomics can be summarized as image acquisition, image reconstruction, image preprocessing, region of interest (ROI) recognition and segmentation, and feature extraction, and predictive models were subsequently constructed using radiomic features combined with disease information.^[^ ^11^, ^12^ ^]^ Therefore, the diagnosis, classification, curative effect, and prognosis of the disease can be predicted and judged based on radiomics.^[^ ^13^, ^14^ ^]^ However, a drawback of radiomics is that the biological interpretability of the features is limited, and a large number of higher‐order features cannot be biologically understood, this is also an urgent problem in the field of radiomics.^[^ ^15^ ^]^

The principle of radiogenomics is that the biomedical image of patients is a comprehensive product based on human DNA, RNA, proteins, metabolites and various kinds of epigenetic modifications, so there is a natural correlation between medical imaging data and biological processes such as molecular pathways of diseases.^[^ ^16^, ^17^, ^18^ ^]^ Radiogenomics endeavors to complement the advantages of genomics and radiomics, which are mainly used in oncology research for five purposes: 1) exploring the correlation between imaging information and biological information, or carrying out biological interpretation for imaging subtypes techniques, 2) conducting virtual biopsy through noninvasive imaging, 3) exploring biological interpretability for radiomic model with genomics, 4) augmenting predictive models with radiomic data to provide additional information and improve prediction accuracy, 5) leveraging genomic information as an alternative to imaging data for validation in cohorts lacking imaging records. Five corresponding workflows are formed (described in detail in Section 3). It is worth noting that traditional information processing methods cannot meet the needs of multi‐omics big data processing, necessitating the adoption of novel data processing strategies to integrate different data sets. Recent advances in machine learning have given impetus to improved model prediction accuracy, facilitated deeper integration of multiple omics, and accelerated clinical transformation in radiogenomics. In addition, we note that the term “Radiogenomics” has also been used to describe a method for identifying genetic variants associated with susceptibility to ionizing radiation,^[^ ^19^ ^]^ and we do not discuss such studies here. In addition, this review will only discuss imaging techniques frequently used in the clinical practice, including CT, MR, ultrasound, and PET, excluding preclinical or early‐phase clinical trial imaging methods pertaining to radiogenomics.

**Figure 1:**
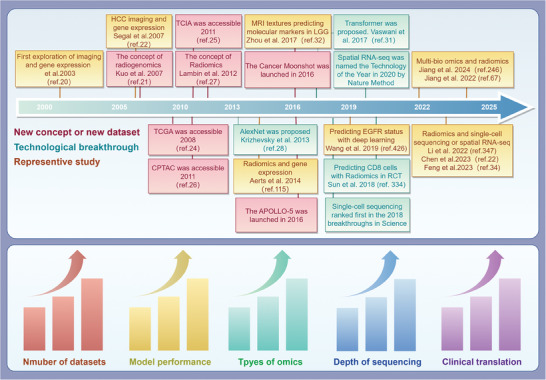
Timeline of the development history of radiogenomics. In this figure, we summarize the emergence of new concepts and databases, breakthroughs in major technologies, and representative studies. Over time, the number of databases has increased, the efficacy of predictive models has improved, the types of omics involved have become diverse, and advancements in sequencing have led to enhanced precision and depth. Furthermore, a growing number of studies have emphasized the clinical translation of radiogenomic research. Created with Figdraw.com (ID: PAAYP8e848).

Radiogenomics is composed of three fields: medical imaging, high‐throughput omics technologies, and artificial intelligence (AI). The development of these three fields has promoted the progress of radiogenomics, therefore, the concept and applications of radiogenomics are constantly evolving and renewing (**Figure** **1**). In 2003, Hobbs et al. sampled the enhanced and non‐enhanced MRI regions of four cases from glioblastoma multiforme patients, and proteomics found that the two regions presented different gene expression profiles.^[^ ^20^ ^]^ In 2007, Kuo et al. used six predefined imaging phenotypes to correlate with tumor microarray data (each microarray can obtain expression information for ≈18 000 genes), and found that gene expression phenotypes associated with doxorubicin drug response could be determined non‐invasively by CT.^[^ ^21^ ^]^ At the same time, they defined and quantified 28 imaging features in hepatocellular carcinoma (HCC), demonstrating that specific gene expression can be mapped to the corresponding imaging features using unsupervised clustering.^[^ ^22^ ^]^ Subsequently, they proposed the original concept of radiogenomics: A methodology for associating gene expression information derived from high‐throughput technologies with radiographic imaging phenotypes.^[^ ^23^ ^]^ However, constrained by the fact that the concept of radiomics had not yet been proposed at that time, their analysis was limited to only a small number of semantic features of the imaging. The Cancer Genome Atlas Program (TCGA) and the matched The Cancer Imaging Archive (TCIA) database has made imaging data, genomic, transcriptomic and proteomic data publicly available from 2008 to 2011.^[^ ^24^, ^25^ ^]^ Similarly, Clinical Proteomic Tumor Analysis Consortium (CPTAC) has also successively released pan‐cancer multi‐omics data including imaging data after 2011,^[^ ^26^ ^]^ meanwhile, Cancer Moonshot Biobank (CMB) and Applied Proteogenomics Organizational Learning and Outcomes (APOLLO‐5) network with pan‐cancer multi‐omics data including imaging data have been started in 2016. Lambin et al. put forward the concept of radiomics in 2012, which can extract high‐throughput information from medical image images^[^ ^27^ ^]^ and has since become a commonly used omics form in radiogenomics. Also in 2012, AlexNet was proposed in the ImageNet Competition, where this deep learning network achieved better classification results than traditional classifiers.^[^ ^28^ ^]^ Since 2013, radiogenomics has published a series of studies that use imaging signatures in conjunction with genomic or transcriptomic data.^[^ ^29^ ^]^ However, since the classifier was not robust enough at the time, researchers often can only get the relationship between biological information and radiological features through correlation analysis,^[^ ^30^ ^]^ so it is difficult to directly use imaging information to predict the outcome of interest. With the development of machine learning models, especially advances in deep learning, such as the Transformer model presented in 2017,^[^ ^31^ ^]^ classifiers now possess the capability to directly predict gene mutations, pathway activation or inhibition, epigenetics changes, and metabolic changes from imaging of tumors.^[^ ^32^ ^]^ More recently, advances in single‐cell technology as well as in spatial sequencing technology^[^ ^33^ ^]^ have made it possible to the jointly analyze imaging and gene expression at the cellular level, or imaging with spatial information of gene expression. Now, scientists have turned their attention to the use of deep learning and multi‐omics data in the context of large cohorts and multicenter data to achieve non‐invasive precision medicine in cancer patients.

Although previous studies have reviewed the advancements in radiomics and radiogenomics, there is still an urgent need for a more updated, comprehensive review that focuses on the streamlining of workflows while encompassing the latest omics technologies. This review aims to summarize the development of radiogenomics and the related omics technologies, and compare the 5 typical workflows of radiogenomics and their applications in various tumors. The application of artificial intelligence technology is highlighted, and the opportunities and challenges brought by tumor heterogeneity are discussed. We also summarize the application of radiogenomics in predicting tumor immune microenvironment, and propose the unique role of radiogenomics in PET. We also discuss the role of radiogenomics in multi‐omics research. Finally, we discussed the challenges faced by clinical transformation, along with future trends in this field.

## 2 Omics Technologies Involved in Radiogenomics

### 2.1 Genomics, Transcriptomics and Proteomics

**Figure 2:**
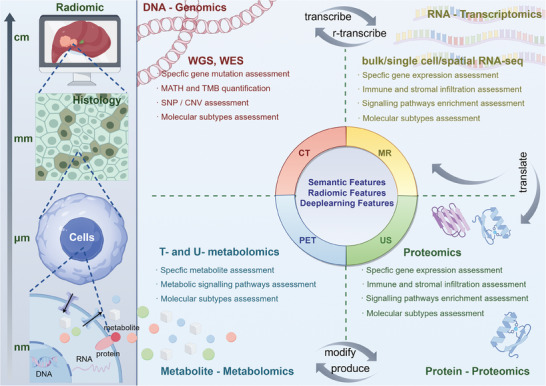
Overview of radiogenomics. In this figure, we illustrate the key elements of radiogenomics including medical imaging, high‐throughput omics technologies, and AI. In addition, we illustrate the relationships among various omics technologies surrounding the central dogma, as well as the subtypes and functions of these technologies. Created with Figdraw.com (ID: PAPOP94688).

Since the advent of Sanger sequencing in 1977,^[^ ^1^ ^]^ genomics has advanced significantly, leading to a rapid decrease in genome sequencing costs due to next‐generation sequencing technologies.^[^ ^34^ ^]^ Genomics technologies, such as whole genome sequencing, whole exome sequencing, and targeted panel sequencing, are widely used in molecular typing, targeted drug selection, and immunotherapy selection.^[^ ^35^, ^36^, ^37^ ^]^ As the most mature omics field, genomics can be applied to various genetic alterations and has played a central role in research since the Human Genome Project's completion in 2003.^[^ ^38^ ^]^ Transcriptomics, which analyzes RNA content, provides a dynamic view of genetic information compared to genomics.^[^ ^39^ ^]^ RNA sequencing (RNA‐seq) and microarrays are two prevalent techniques for transcriptomic analysis, with RNA‐seq offering more accurate and sensitive RNA quantification.^[^ ^40^, ^41^ ^]^ Proteomics, focusing on proteins and their post‐translational modifications, addresses the limitations of genomics and transcriptomics by providing unique information on gene expression.^[^ ^42^ ^]^ The Human Proteome Project aims to chart the complete human proteome using advanced proteomic technologies.^[^ ^43^ ^]^ Mass spectrometry‐based proteomics allows for large‐scale detection and comprehensive analysis of protein information.^[^ ^44^ ^]^ Both transcriptomics and proteomics can quantify gene expression in tumors, immune and stromal cell infiltration, determine pathway activation or inhibition, and evaluate molecular subtypes^[^ ^45^, ^46^ ^]^ (**Figure** **2**).

### 2.2 Metabolomics, Microbiomics and Epigenetic Modifications (omics)

Metabolomics, a new omics direction utilizing techniques like nuclear magnetic resonance and chromatography‐mass spectrometry, is divided into targeted and untargeted approaches to identify and quantify metabolites.^[^ ^47^ ^]^ It detects various substances, including amino acids, carbohydrates, nucleotides, and lipids, and is increasingly applied in tumor research due to metabolic reprogramming being a tumor hallmark. Metabolites provide additional information on disease heterogeneity and cancer prognosis.^[^ ^48^ ^]^ Microbiomics, the study of all microorganisms and their genetic information in an environment, employs metagenomic and 16s rRNA gene sequencing, revealing correlations between microorganisms and tumor phenotypes.^[^ ^49^ ^]^ Epigenetic modifications, encompassing DNA, RNA, and protein modifications, are variable and can alter gene expression, affecting tumor phenotypes.^[^ ^50^, ^51^ ^]^ Joint analysis of imaging with metabolomics, microbiomics, and epigenetic modifications presents promising avenues for research.

### 2.3 Single‐Cell Sequencing and Spatial Sequencing

Single‐cell sequencing, which encompasses genomics, transcriptomics, proteomics, and metabolomics at the individual cell level, can be categorized into single‐cell DNA sequencing, single‐cell RNA sequencing (scRNA‐seq), single‐cell proteomics, and single‐cell metabolomics.^[^ ^52^, ^53^, ^54^ ^]^ ScRNA‐seq is the most prevalent, enabling the analysis of gene expression heterogeneity across various cell types within tissues, unlike bulk RNA‐seq that only considers average gene expression.^[^ ^55^ ^]^ ScRNA‐seq also facilitates the study of cell grouping, differentiation, and the relationship between specific genes and cell populations.^[^ ^56^, ^57^ ^]^ However, both bulk and single‐cell sequencing methods can lose spatial information.^[^ ^58^ ^]^ To address this, spatial sequencing, particularly spatial transcriptomics, has emerged. By integrating tissue section localization with transcriptome sequencing, spatial transcriptomics reveals the spatial distribution of gene expression and microenvironments distinguished by unique gene signatures.^[^ ^59^ ^]^ Combining scRNA‐seq with spatial transcriptomics allows precise mapping of transcriptionally annotated single cells within their native tissue frameworks, enhancing our understanding of cellular interactions in development, physiological balance, and disease states.^[^ ^60^ ^]^

### 2.4 Radiomics

The definition of radiomics is to extract a large number of features from radiological images with high throughput, use automatic or semi‐automatic analysis methods to convert imaging data into quantitative features, and then mine the relationship between these quantitative features and disease stage, grade as well as prognosis of patients.^[^ ^27^, ^61^ ^]^ The definition of radiomics originated from radiogenomics, and its idea originated from the heterogeneity of tumors. The specific implementation methods of radiomics can be simply summarized as follows: 1) collect medical imaging such as CT, MR, PET or ultrasound. 2) preprocess or standardize medical imaging. 3) segment the ROI within the medical images, either manually, semi‐automatically, or fully automatically. 4) extract the radiomic features. 5) build predictive models or correlation maps. Compared to other omics technologies, the advantage of radiomics is that high‐dimensional quantitative features can be obtained without tissue samples to support the medical decision‐making process.^[^ ^62^ ^]^ Other omics can directly analyze the molecular components within biological organisms, providing a more direct insight into alterations in gene expression, protein function, and metabolic activities in tumors. Radiomics and other omics technologies complement each other, enabling a transition from invasive biopsies to virtual biopsies. It adds biological interpretability to otherwise inexplicable radiomic predictive models. The joint application of radiomics with other omics technologies will be discussed in detail in Sections 3 and 9.

## 3 Typical Workflows and Available Public Databases of Radiogenomics

### 3.1 Workflow 1‐Correlation Analysis (CA)

**Figure 3:**
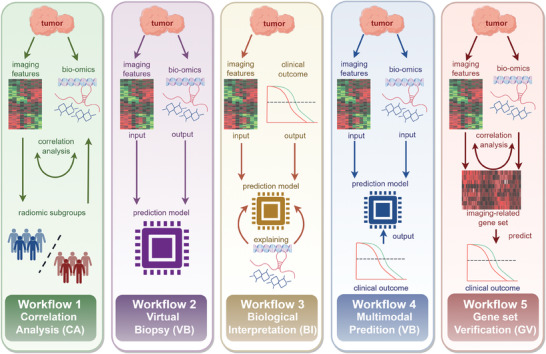
Typical workflows of radiogenomics. Combinations of medical imaging, high‐throughput omics technologies, and AI formulate 5 typical workflows including workflow 1‐Correlation Analysis (CA), workflow 2‐Virtual Biopsy (VB), workflow 3‐Biological Interpretation (BI), workflow 4‐Multimodal Prediction (MP) and workflow 5‐Gene Set Verification (GV). Created with Figdraw.com (ID: WOARO7be2f).

Workflow 1‐CA can be summarized as follows: conducting correlation analysis between semantic features, radiomic features, or radiomic subtypes and gene mutation, gene expression, or molecular subtype (**Figure** **3**). Or conducting biological interpretation for specific imaging subtypes. This workflow is the earliest application in radiogenomics. Its main purpose is to explore the correlation between imaging features and gene mutations or expressions and to suggest the biological basis behind imaging features or imaging subtypes. In this process, the construction of prediction models is often not involved. For example, Karlo et al. extracted 8 qualitative and 5 quantitative imaging features from 233 patients with clear cell renal cell carcinoma (ccRCC) and extracted the mutation information of VHL, PBRM1, BAP1, SETD2, and KDM5C from the genomics. Correlation analysis revealed that these gene mutations were significantly associated with some imaging features.^[^ ^29^ ^]^ Zhou et al extracted 87 semantic features from 113 patients with non‐small cell lung cancer (NSCLC) and performed RNA‐seq of tumor tissues. The results showed that there were 32 significant correlations between semantic features and metagene.^[^ ^63^ ^]^ From a clinical application perspective, workflow CA often conducts preliminary exploratory research, and additional workflows are required to establish predictive models to guide clinical practice.

### 3.2 Workflow 2‐Virtual Biopsy (VB)

Workflow 2‐VB can be summarized as follows: predicting gene mutation, gene expression, or molecular subtypes through semantic features or radiomic features. This workflow is the most widely used, and its main purpose is to conduct virtual biopsy through noninvasive means. Chen et al. extracted 841 radiomic features from glioma MRI images to predict intratumoral M2 macrophages and found that this model could assist in treatment decisions in immunotherapy cohorts.^[^ ^64^ ^]^ Bourbonne et al. extracted 1332 radiomic features from PET/CT images of lung cancer patients to predict KEAP1/NFE2L2 mutations, and the prediction model can stratify the outcomes of external cohort receiving radiotherapy.^[^ ^65^ ^]^ Compared to workflow CA, workflow VB demonstrates a more pronounced goal‐orientation in clinical applications. It tends to collect high‐dimensional data (such as radiomic data) and strives for more precise predictive outcomes by employing data dimensionality reduction techniques and high‐performance classifiers.

### 3.3 Workflow 3‐Biological Interpretation (BI)

Workflow 3‐BI can be summarized as follows: constructing the prediction model of treatment efficacy, prognosis (survival, recurrence, or progression), or biomarker for cancer patients based on the semantic features or radiomic features, and then using other omics to biologically interpret the prediction model. The main purpose of workflow BI is to understand the biological basis behind the prediction model. This is because when designing the imaging prediction model, the output of the model is often the outcomes of clinical concern (such as survival, or treatment response), which makes the model difficult to interpret without any biological hypothesis. In 169 imaging‐pathology paired patients with HCC, Feng et al. constructed a radiomic model to predict the macrotrabecular‐massive subtype of HCC, and the biological interpretation using bulk RNA‐seq, scRNA‐seq as well as the spatial transcriptome revealed that the predictive model was associated with humoral immunodeficiency.^[^ ^66^ ^]^ Jiang et al. constructed a single radiomic feature model that predicted the survival of patients with triple‐negative breast cancer, then they used transcriptomics and metabolomics finding that the model was associated with fatty acid metabolism alterations in tumors.^[^ ^67^ ^]^

### 3.4 Workflow 4‐Multimodal Prediction (MP)

Workflow 4‐MP can be summarized as follows: genomic information, imaging information, or other clinical information jointly build the predictive model to predict the efficacy of treatment, prognosis (survival, recurrence, or progression), or biomarkers. The main purpose of workflow MP is to build higher dimensional data by combining the macroscopic imaging information with the biological information brought by the microscopic genomics to improve the prediction accuracy. Kickingerder et al. found that integrating the radiomic model into the molecular predictive model can significantly improve the accuracy of the original model in predicting the survival of glioma patients.^[^ ^68^ ^]^ Yi et al. used pre‐treatment CT images of ovarian cancer, clinical characteristics, and single nucleotide polymorphism information (genomics of blood) to jointly construct a platinum drug resistance predictive model, and the model constructed by the combination of the three achieved better prediction accuracy than the single information construction model.^[^ ^69^ ^]^ It is noteworthy that the distinctiveness of workflow MP lies in its emphasis on integrating multimodal data, including genomics, imaging, and clinical information, to build a comprehensive predictive model for joint prediction of clinical outcomes. This approach contrasts with workflow VB and workflow BI, which typically rely solely on imaging information to construct their predictive models.

### 3.5 Workflow 5‐Gene Set Verification (GV)

Workflow 5‐GV can be summarized as finding the characteristic gene set of semantic features, radiomic features, or imaging subtypes through correlation analysis (such as weighted correlation network analysis), and using the characteristic gene set to verify the survival stratification in the cohort without imaging information. Replacing imaging features with characteristic gene sets can not only verify the biological background of imaging features but also expand the sample size of the total cohort. Wu et al. found 73 genes with a strong correlation with tumor adjacent parenchymal imaging feature through weighted correlation network analysis and used these 73 genes to predict this imaging feature, which was verified in the dataset without imaging data.^[^ ^70^ ^]^ Using Fluorodeoxyglucose (FDG)‐PET parameters as the gold standard, Choi et al. constructed the tumor metabolism index using transcriptomic data and performed a survival analysis using tumor metabolism index in cohorts without PET data.^[^ ^71^ ^]^ Similarly, An et al. explored the biological basis of FDG uptake in patients with HCC and constructed a functional gene set associated with FDG uptake finding that patients with high FDG uptake had higher HCC recurrence rates.^[^ ^72^ ^]^ Currently, in publicly available tumor sequencing databases, only a small proportion of the data is accompanied by imaging information. By employing the workflow GV method, we can construct gene sets that are closely related to imaging and further validate them extensively across numerous public databases. This approach will significantly enhance the credibility and persuasiveness of studies. Although there are currently few studies utilizing workflow GV, it is foreseeable that the application of this method will become increasingly widespread in the future.

### 3.6 Image‐Gene Datasets That Have Been or Will be Publicly Available

At present, most of the image‐gene datasets that have been publicly available or will be publicly available can be accessed through TCIA (https://dev.cancerimagingarchive.net/). The pan‐cancer imaging data of TCGA and CPTAC can be accessed directly through TCIA. Their clinical information or genomic, transcriptomic, and proteomic data need to be downloaded through the official website. APOLLO‐5 and CMB are also collecting pan‐cancer data. In addition, the NSCLC radiomics genomics and NSCLC radiomics datasets disclose the imaging data, clinical data, and their corresponding transcriptomic information of patients with NSCLC. The glioma imaging data of the Chinese glioma Genome Atlas (CGGA) dataset requires an application to obtain access to the data. The glioma imaging data of IvyGAP and REMBRANDT can be accessed directly through TCIA, and the corresponding sequencing data need to be obtained on the official website. Relevant dataset information and webpages can be obtained in **Table** **1**.

**Table 1:** Available public databases of radiogenomics.

Types of tumors	Open‐source database	Main Imaging modality	Types of biotechnology	Website	Status
Pan‐cancers	TCGA	–	Genomics, transcriptomics and proteomics	https://www.cancerimagingarchive.net/collection/tcga‐lusc/ (LUSC for example)	Complete
	CPTAC	–	Genomics, transcriptomics and proteomics	https://www.cancerimagingarchive.net/collection/cptac‐luad/ (LUAD for example)	Ongoing
	APOLLO‐5	–	Genomics, transcriptomics and proteomics	https://www.cancerimagingarchive.net/collection/apollo‐5/	Ongoing
	CMB	–	Genomics, transcriptomics and proteomics	https://www.cancerimagingarchive.net/collection/cmb‐lca/ (Lung Cancer for example)	Ongoing
Lung cancer	NSCLC‐Radiomics‐Genomics	CT	Transcriptomics	https://www.cancerimagingarchive.net/collection/nsclc‐radiomics‐genomics/	Complete
	NSCLC Radiogenomics	PT, CT	Transcriptomics	https://www.cancerimagingarchive.net/collection/nsclc‐radiogenomics/	Complete
Glioma	CGGA	MRI	Genomics and transcriptomics	http://www.cgga.org.cn/	Ongoing
	IvyGAP	MRI	Transcriptomics	https://www.cancerimagingarchive.net/collection/ivygap/	Complete
	REMBRANDT	MRI	Genomics and transcriptomics	https://www.cancerimagingarchive.net/collection/rembrandt/	Complete
	GBMatch	MRI	Genomics	https://medical‐epigenomics.org/papers/GBMatch/	Complete

Note. TCGA: The Cancer Genome Atlas Program; CPTAC: Clinical Proteomic Tumor Analysis Consortium; APOLLO: Applied Proteogenomics Organizational Learning and Outcomes; CMB: The Cancer Moonshot Biobank; LUSC: Lung Squamous Cell Carcinoma; LUAD: Lung Adenocarcinoma; NSCLC: Non‐small‐cell lung cancer; CGGA: Chinese Glioma Genome Atlas; IvyGAP: Ivy Glioblastoma Atlas Project; REMBRANDT: REpository for Molecular BRAin Neoplasia DaTa.

## 4 Using five Workflows to Interpret Radiogenomics Studies of Various Tumors

### 4.1 Liver Cancer, Colorectal Cancer, and Other Gastrointestinal Tumors

The main types of primary liver cancer include HCC, intrahepatic cholangiocarcinoma, and mixed differentiated carcinoma. HCC is highly heterogeneous at the genomic and histological levels, resulting in the lack of molecular typing‐based treatment options.^[^ ^73^, ^74^ ^]^ Despite the tremendous progress made in recent years with molecularly targeted therapies combined with immunotherapy, patient survival remains poor.^[^ ^75^ ^]^ Under the paradigm of workflow CA, some studies have found that LI‐RADS are correlated with fractional allelic imbalance rate index or high‐frequency mutations,^[^ ^76^, ^77^ ^]^ and some studies have analyzed the correlation between image semantic features and gene expression.^[^ ^21^, ^22^, ^78^, ^79^, ^80^ ^]^ In addition, Gu et al. constructed two types of radiomic subtypes using similarity network fusion and used transcriptomics to biologically interpret the radiomic subtypes.^[^ ^81^ ^]^ Under the paradigm of workflow VB, eight image features can be used to predict IDH mutations in intrahepatic cholangiocarcinoma.^[^ ^82^ ^]^ Similarly, radiomic features can also predict the PI3K signaling pathway in HCC.^[^ ^83^ ^]^ Using workflow BI, after establishing the imaging‐clinical outcome model^[^ ^84^ ^]^ or imaging‐pathology model,^[^ ^66^ ^]^ transcriptomics was used to carry out the relevant biological interpretation of these predictive models.

The incidence and mortality of colorectal cancer are increasing. Although survival can be improved by targeted therapy or immunotherapy, recurrence, and metastasis remain important causes of death in patients.^[^ ^85^, ^86^ ^]^ Applying workflow CA, BRAF mutations have been found to be associated with texture features^[^ ^87^ ^]^ and KRAS mutations have been found to be associated with texture features.^[^ ^88^ ^]^ Applying workflow VB, imaging information can be used to predict TP53 status as well as KRAS mutation status.^[^ ^89^, ^90^, ^91^ ^]^

Other gastrointestinal tumors include esophageal cancer, gastric cancer, and pancreatic cancer. Applying workflow CA, studies have reported that imaging information is associated with gene mutations^[^ ^92^ ^]^ or gene expression profiles^[^ ^93^ ^]^ in pancreatic cancer. Similarly, studies have reported that imaging information is associated with gene expression in esophageal cancer.^[^ ^94^ ^]^ Applying workflow VB, ITGAV expression,^[^ ^95^ ^]^ SMAD4 status, and tumor stromal content of pancreatic cancer could be predicted using imaging information.^[^ ^96^ ^]^ Modeling using imaging information can also predict chromosomal instability status, and HER2 status in gastric cancer.^[^ ^97^, ^98^ ^]^ Applying workflow BI, an imaging‐treatment response model was constructed in esophageal cancer and the results of the model were found to be associated with the extracellular matrix and WNT signaling pathway.^[^ ^99^ ^]^ Similarly, radiomic models were constructed to predict the survival of patients with gastric cancer and were biologically interpreted by transcriptomics.^[^ ^100^, ^101^, ^102^ ^]^ Workflow MP was applied, combining genomics and imaging information to improve the stratification of progression‐free survival or disease‐free survival in patients with esophageal cancer.^[^ ^103^, ^104^ ^]^

### 4.2 Lung Cancer

Lung cancer is one of the leading causes of cancer‐related death and the second most common tumor with reported morbidity and mortality of ≈11.4% and 18%, respectively.^[^ ^86^ ^]^ Advances in targeted therapies and immunotherapy have greatly improved survival and quality of life in patients with lung cancer,^[^ ^105^, ^106^ ^]^ but both treatments often require a clear histological sample by immunohistochemistry or next‐generation sequencing (NGS) to determine their target or indication.^[^ ^107^, ^108^ ^]^ Therefore, the imaging‐gene relationship can be explored through the various workflows of radiogenomics to make efforts for clinical transformation. Workflow CA showed that EGFR mutation,^[^ ^109^, ^110^ ^]^ KRS mutation,^[^ ^109^, ^111^ ^]^ ALK mutation,^[^ ^109^, ^112^ ^]^ and ROS1 mutation^[^ ^113^ ^]^ were correlated with imaging features. Gene expression profiles can also be reflected from imaging information.^[^ ^63^, ^114^, ^115^, ^116^, ^117^, ^118^ ^]^ In addition, the broad‐based NGS of Phase 1 lung adenocarcinoma allows for the biological interpretation of the radiomic subtypes identified by consensus clustering.^[^ ^119^ ^]^ Workflow VB is widely used, using semantic features or imaging to predict EGFR mutations,^[^ ^120^, ^121^, ^122^, ^123^, ^124^, ^125^, ^126^, ^127^, ^128^, ^129^ ^]^ KRS mutations,^[^ ^111^, ^120^, ^127^, ^128^, ^129^, ^130^, ^131^ ^]^ and ALK mutations^[^ ^132^, ^133^, ^134^ ^]^ in lung cancer may play a role in supporting decision‐making for targeted therapies. An imaging‐clinical outcome prediction model was constructed around workflow BI, and transcriptomics^[^ ^135^, ^136^, ^137^, ^138^ ^]^ was utilized for the biological interpretation of the model. Applying workflow MP, combining genomics or transcriptomics with imaging models to form radiogenomics prediction models, the ability of the model to predict clinical treatment outcomes^[^ ^133^, ^139^, ^140^, ^141^, ^142^ ^]^ or biomarkers^[^ ^143^ ^]^ was improved. Finally, in the model of workflow GV, Lee et al. constructed an imaging‐related gene set for the semantic feature of lung cancer‐pleural contact index and extended this gene set to a cohort without imaging, the prognostic performance of a gene substitute for pleural exposure index was validated.^[^ ^139^ ^]^

### 4.3 RCC and Other Urinary Tumors

RCC ranks among the top ten common tumors in the world, with ccRCC being its primary subtype, accounting for ≈75% of all renal cancers.^[^ ^144^ ^]^ ccRCC is a very lethal tumor, although nephrectomy offering a potential cure, 30% of patients with limited stage will develop metastasis.^[^ ^145^ ^]^ Given the unique angiogenic profile of ccRCC, altered immune microenvironment, and programmed death protein 1 ligand expression profile,^[^ ^146^ ^]^ anti‐vascular agents as well as immunotherapy have been developed, and these therapies are expected to improve overall survival.^[^ ^147^, ^148^, ^149^ ^]^ However, at present, only some patients respond to these therapies, emphasizing the urgent need for more precise methods to understand the molecular heterogeneity of each ccRCC to further assist treatment decisions. Applying workflow CA, imaging features were found to be correlated with gene mutations^[^ ^150^, ^151^ ^]^ or gene expression profiles.^[^ ^29^, ^150^, ^152^, ^153^, ^154^, ^155^, ^156^, ^157^, ^158^ ^]^ In addition, transcriptomics can be used to biologically interpret the imaging subtypes of ccRCC.^[^ ^159^ ^]^ Using workflow VB, radiomic or semantic features can predict gene mutation,^[^ ^160^, ^161^, ^162^ ^]^ DNA methylation,^[^ ^163^, ^164^ ^]^ gene expression pattern,^[^ ^165^, ^166^ ^]^ and lipid metabolism of ccRCC.^[^ ^167^ ^]^ Applying workflow BI to biologically interpret the constructed imaging‐clinical outcome predictive model helps us understand the biological process and molecular mechanism of ccRCC.^[^ ^168^, ^169^, ^170^, ^171^ ^]^

Other urinary tumors involved in radiogenomics include bladder and prostate cancer. Applying workflow CA, imaging features of prostate cancer were associated with gene mutations^[^ ^172^ ^]^ or gene expression profiles.^[^ ^173^, ^174^, ^175^, ^176^ ^]^ Applying workflow VB, m6a score for bladder cancer^[^ ^177^ ^]^ as well as angiogenesis^[^ ^178^ ^]^ can be predicted by imaging information, as can predict hypoxia status^[^ ^179^ ^]^ and Decipher score of prostate cancer.^[^ ^180^ ^]^ Using workflow BI, the imaging‐invasiveness prediction model of prostate cancer^[^ ^181^ ^]^ and the imaging‐survival prediction model of bladder cancer^[^ ^182^ ^]^ can be biologically interpreted. Using workflow MP, the combination of transcriptomics and imaging information improved the accuracy of predicting the staging of bladder cancer.^[^ ^183^ ^]^

### 4.4 Glioma

Glioma is a malignant tumor derived from glial cells, represents the most prevalent cancer within the central nervous system, characterized by a high recurrence rate and poor prognosis.^[^ ^184^ ^]^ Applying workflow CA can reveal the relationship between semantic features and gene expression,^[^ ^185^, ^186^, ^187^, ^188^, ^189^ ^]^ and workflow VB can be used to build predictive models to predict IDH mutations,^[^ ^32^, ^190^, ^191^, ^192^, ^193^, ^194^ ^]^ 1p/19q co‐deletion,^[^ ^32^, ^192^, ^193^, ^194^, ^195^, ^196^, ^197^ ^]^ O_6_ methylguanine‐DNA‐methyltransferase gene promoter status,^[^ ^191^, ^194^, ^198^, ^199^, ^200^, ^201^ ^]^ H3K27M mutation,^[^ ^202^, ^203^ ^]^ medulloblastoma subgroups,^[^ ^204^, ^205^ ^]^ MYCN amplification,^[^ ^206^, ^207^ ^]^ CIC mutation status,^[^ ^208^ ^]^ COC molecular subtypes.^[^ ^209^ ^]^ Workflow BI was applied to build an imaging‐prognosis prediction model, and transcriptome was used for biological interpretation.^[^ ^210^, ^211^, ^212^, ^213^, ^214^, ^215^, ^216^ ^]^ Applying workflow MP, the fusion of radiomic information and genomic information could improve the prediction of patient survival.^[^ ^217^, ^218^ ^]^ Applying workflow GV, relevant gene sets were constructed for imaging‐clinical outcomes and validated in cohorts without imaging data.^[^ ^211^, ^219^ ^]^

### 4.5 Breast Cancer

Breast cancer is the most common malignancy in women and has a high mortality rate.^[^ ^86^ ^]^ Given its intricate pathogenesis, often entailing a variety of genetic alterations,^[^ ^220^ ^]^ radiogenomics emerges as a pivotal tool to carry out high‐quality precision treatment, which is essential to improve the survival time and quality of life of patients. Applying workflow CA, it was found that imaging information was related to gene expression modules^[^ ^70^, ^221^, ^222^, ^223^, ^224^, ^225^, ^226^, ^227^, ^228^, ^229^ ^]^ or gene mutations.^[^ ^230^, ^231^ ^]^ Applying workflow VB, imaging models were constructed to predict Oncotype DX,^[^ ^224^, ^232^, ^233^ ^]^ molecular types,^[^ ^234^, ^235^, ^236^, ^237^ ^]^ and expression levels of specific genes or gene expression modules.^[^ ^238^, ^239^, ^240^ ^]^ Apply workflow BI, using transcriptomics^[^ ^67^, ^241^, ^242^, ^243^ ^]^ or proteomics^[^ ^244^ ^]^ to biologically interpret the imaging clinical results. Applying workflow MP, we can better predict the recurrence,^[^ ^245^ ^]^ treatment response,^[^ ^246^ ^]^ lymph node metastasis,^[^ ^242^, ^247^ ^]^ or survival^[^ ^248^ ^]^ of breast cancer.

### 4.6 Other Tumors

Due to the incidence or imaging information acquisition, there are relatively few studies involving radiogenomics in other tumors. Other cancers such as ovarian cancer, melanoma, head and neck cancer, and gastrointestinal stromal tumors have also been preliminarily explored. Applying workflow CA, imaging features were found to be associated with specific gene mutation status of head and neck cancer^[^ ^249^, ^250^ ^]^ or ovarian cancer^[^ ^251^, ^252^ ^]^ Using workflow VB, imaging models were constructed to predict the BRAF status of melanoma brain metastasis,^[^ ^253^ ^]^ hypoxia of ovarian cancer,^[^ ^254^ ^]^ and KIT gene mutations of gastrointestinal stromal tumors.^[^ ^255^, ^256^, ^257^ ^]^ Table S1 (Supporting Information) presents the specific details of these typical radiogenomic studies.

## 5 AI is An Important Part of Radiogenomics

### 5.1 Scope and Development of AI

AI emerged in the mid‐1950s.^[^ ^258^ ^]^ AI is a scientific and engineering field that studies how to enable computer systems to perform tasks requiring human intelligence, with the primary goal of simulating and replicating all aspects of human intelligence, thereby allowing computer systems to perform cognitive and problem‐solving tasks similar to those of humans. AI primarily relies on learning from data, but also on prior knowledge and rule systems.^[^ ^259^, ^260^ ^]^ As a branch of AI, machine learning flourished in the 1980s.^[^ ^261^ ^]^ Machine learning focuses on how to improve the performance of computer systems by learning patterns and rules from data without explicit programming. The purpose is to enable machines to acquire knowledge from users and input data, so that they can automatically make judgments and responses in the actual environment of production and life, so as to help us solve more problems, reduce errors, and improve efficiency. Machine learning includes supervised learning, unsupervised learning, semi‐supervised learning, and reinforcement learning.^[^ ^262^ ^]^ As a special form and new direction of machine learning, deep learning is developing rapidly nowadays, and its introduction has propelled AI to a new climax. Deep learning uses neural networks to enhance the expression of complex tasks, allowing machines to find feature extraction methods automatically through neural networks.^[^ ^263^ ^]^

### 5.2 Image Segmentation Based on AI

Similar to radiomics, automatic segmentation using AI also plays an important role in radiogenomics, because except for end‐to‐end models, medical images need to be segmented before feature extraction, that is, depicting tumor boundaries. Early medical image segmentation algorithms predominantly relied on traditional methods, including the edge detection filter and other methods.^[^ ^264^ ^]^ Nonetheless, the complexity of medical images and the instability of segmentation results restrict the development of these algorithms in medical image segmentation. The subsequent image segmentation is still mainly completed by professional radiologists. However, the emergence of AI has made it possible to segment annotations automatically. Machine learning or deep learning models can be trained for labels by a part of manually sketched tumor boundaries, which greatly reduces the time cost and professional knowledge requirements required for annotation. Deep learning‐based segmentation algorithms are the most common algorithms in medical image segmentation, such as the fully convolutional neural network (FCN) and U‐net network. FCN, a special type of neural network structure, improves the image segmentation accuracy by replacing the full connection layer with the convolution or upsampling layer, using deconvolution to sample on the output feature map, and replacing the convolutional neural network for image classification with a dense prediction network for image segmentation.^[^ ^265^ ^]^ Ben‐cohen et al. first used FCN to segment medical images, and they used FCN to complete the tasks of CT liver image segmentation and liver metastasis detection.^[^ ^266^ ^]^ It is worth noting that FCN can accept inputs of any size, thereby eliminating redundant computation of the network and achieving results close to manual segmentation. Similarly, Sun et al. also developed a fully automatic liver tumor CT image segmentation method based on FCN, and achieved high accuracy and robustness.^[^ ^267^ ^]^ The U‐net network is an image segmentation network based on a symmetric encoder‐decoder structure of FCN. It consists of an encoder (subsampling path) and a decoder (upsampling path). Its shape resembles a U‐shape, hence the name U‐net. The U‐net combines feature maps in the encoder with feature maps in the decoder via skip connections to retain more positional information and improve segmentation accuracy. The U‐net has achieved remarkable results in medical image segmentation, accurately segmenting tumors, brain structures and other regions. Mahajan et al. used a standard 3D U‐net architecture to complete the image segmentation task, thus ultimately helping to build a radiogenomics model to complete the mutation prediction of EGFR.^[^ ^123^ ^]^ Similarly, Kihira et al. designed a U‐net framework based on deep learning using a symmetric structure,^[^ ^191^ ^]^ and tried various U‐net methods: ResNet50 and DenseNet121 networks pre‐trained with ImageNet and RadimageNet, respectively, and achieved a high dice similarity coefficient on the prediction set, of which the DSCs of DenseNet121 networks pre‐trained with RadimageNet reached 0.93. In addition, in the radiogenomics study of brain tumors by Bakas et al.^[^ ^268^ ^]^ and Sayah et al.,^[^ ^269^ ^]^ the GLISTRBoost tool was applied to automatically segment tumor lesions. GLISTRBoost is an automatic segmentation tool specially applied to glioma, which is used to generate segmentation labels. It won the championship in the 2015 international multimodal brain tumor image segmentation challenge.^[^ ^270^ ^]^ The tool uses the Expectation‐Maximization framework to map each sub‐region of the brain scan automatically, and finally classifies the voxels of brain tissue into four categories: enhanced tumor, necrotic non‐enhanced core, peritumoral edema, and normal brain tissue.

### 5.3 Image Feature Extraction, Feature Selection and Dimensionality Reduction Based on AI

The core step of radiogenomics is to extract high‐throughput features to quantitatively analyze the attributes of segmented lesions. Features generally include traditional radiomic features and depth features based on CNN. The former usually includes first‐order features, second‐order features, high‐order features, shape, and size. To describe the data more comprehensively, a large number of features are usually generated from many aspects during feature extraction. However, too many features are not necessarily conducive to the analysis of the results. Because a large number of features inevitably have features that are not relevant to the clinical problem, and sufficient data is typically required to support the robustness and accuracy of the model, the over‐fitting of the model usually occurs when the number of features is large and the number of data is small.^[^ ^271^ ^]^ Similarly, high‐dimensional data from genomics need to be filtered and dimensionalized for further downstream analysis. Therefore, feature screening and dimensionality reduction is an essential part of radiogenomics.

Commonly used feature filtering dimensionality reduction methods include embedded method: such as least absolute shrinkage and selection operation (LASSO), wrapped method: such as recursive feature elimination, filtered method: such as Chi‐square test, correlation coefficient, maximum correlation, and minimum redundancy (mRMR), and machine learning models: such as support vector machines (SVM), and random forests (RF). LASSO, as a classical linear regression algorithm, is widely used in feature selection and dimension reduction.^[^ ^272^ ^]^ Compared with the traditional linear regression algorithm, the LASSO algorithm can automatically screen out the characteristic variables that have a greater impact on the target variables while maintaining the prediction accuracy, to reduce the complexity of the model and improve the generalization performance. In a recent radiogenomic study of ccRCC, He et al. determined the prognostic lipid metabolism related genomic features by using univariate Cox regression and LASSO regression analysis, and screened the imaging features using the mRMR algorithm and LASSO regression.^[^ ^167^ ^]^ They screened 13 gene features related to the prognosis from 776 lipid metabolism related genes, screened 289 features from the initial 1316 imaging features using the mRMR algorithm, and finally retained 9 best features through LASSO regression. The radiogenomics model was constructed from the above 13 gene features and 9 image features screened by dimension reduction. Unlike LASSO regression, the mRMR algorithm belongs to the filtering method. Its goal is to select the most useful subset for the learning task from a set of features, while ensuring the minimum redundancy between these features, that is, maintaining the correlation between features and target variables, reducing the interdependence between features. This method is usually used for data sets with higher dimensions (such as gene sets), which can effectively reduce the number of features, improve the generalization ability of the model and reduce the risk of over‐fitting. Wu et al. used the mRMR algorithm to evaluate the correlation and redundancy of each feature,^[^ ^207^ ^]^ and finally retained the features with high correlation and low redundancy to help build a radiogenomics model to predict MYCN amplification. The same algorithm appeared in the study of Shiri et al.^[^ ^127^ ^]^ and Le et al.^[^ ^128^ ^]^

Compared to traditional algorithms, deep learning has more efficient feature screening and dimensionality reduction capabilities and higher accuracy. For example, CNN, a special type of neural network, is mainly used to process data such as images, videos and text.^[^ ^273^ ^]^ The convolutional layer is the core component of the CNN. It performs convolution operations on the input data using convolution kernels to extract local features. Different convolution kernels can extract different types of features, such as edges, textures, shapes, etc. By stacking multiple convolution layers, more complex and abstract features can be progressively extracted. The output feature map obtained by the convolution layer is input to the pooling layer. The data dimension can be reduced by subsampling operations, which mainly include max pooling and average pooling. Max pooling selects the maximum value in the pooling window as the output, while average pooling calculates the average value of all values in the pooling window as the output. In addition, it is also possible to use a 1*1 convolution kernel with fewer channels than the original number of channels for convolution operations, thereby reducing the number of channels in the feature map. CNN extracts feature information from input data through convolutional layers and implements dimensionality reduction operations through structures such as pooling layers and 1*1 convolutional kernels. These operations make CNN efficient and accurate when processing large, high‐dimensional image or text data.^[^ ^274^, ^275^ ^]^ At the same time, feature extraction and dimensionality reduction methods based on architectures such as CNN, transformer and GAN continue to evolve and innovate, providing more powerful tools for solving complex artificial intelligence problems.

### 5.4 Construction of Prediction Models Based on AI

Modeling analysis is another core part of radiogenomics workflows, which mainly includes data set partitioning, classifier selection and model optimization. The performance of the model is usually closely related to the correct selection of the classifier. It is necessary to select the appropriate machine learning algorithm according to the nature of the problem to be solved and the amount of data. Common classifiers include logistic regression, RF, SVM, and K‐nearest neighbors. Logistic regression, as a generalized linear regression model, is widely used to solve the dichotomous problem in radiogenomics. Saidak et al. constructed a radiogenomics model based on logistic regression to predict vascular complications and coagulation function in patients with glioma.^[^ ^276^ ^]^ As mentioned above, RF is an integrated learning method based on decision tree. Compared with logistic regression, RF can effectively prevent overfitting while ensuring better classification performance. Recently, in a radiogenomics analysis based on the cellular tumor‐stroma heterogeneity of breast cancer, researchers established radiogenomic features to predict the prognosis of breast cancer patients by correlating imaging data with the tumor‐stroma heterogeneity score using an RF classifier.^[^ ^277^ ^]^ For studies with a small amount of data, SVM and K‐nearest neighbors are more suitable. For example, SVM relies on support vectors rather than the whole dataset, so it is more applicable in small sample datasets. At the same time, the decision function of SVM is only determined by a few support vectors, which helps to avoid overfitting and improve the generalizability of the model. Considering the advantages and disadvantages of each classifier model, it seems that building an ensemble machine‐learning model is an option that can be considered. For example, Jin et al. established a superimposed integrated radiogenomics model to predict the expression status of homeodomain‐only protein homeobox.^[^ ^278^ ^]^ Specifically, their model includes two levels. The first level is SVM, RF and gradient‐boosted decision tree, and the predicted value of the first level is combined as the new input variable of the second level model. For deep learning, after data pre‐processing, we can select the appropriate neural network architecture according to the type of task, and use the deep learning framework to build the model. The model is then trained through repeated forward propagation and back propagation, and the model is evaluated and adjusted to improve the model's performance.

## 6 Tumor Heterogeneity is Both a Challenge and an Opportunity

### 6.1 Biological Concept of Tumor Heterogeneity

Tumor heterogeneity stems from the concept that no two tumors are exactly alike^[^ ^279^ ^]^ and can be divided into inter‐tumor heterogeneity as well as intra‐tumor heterogeneity.^[^ ^280^ ^]^ Inter‐tumor heterogeneity is mainly considered from the perspective of bulk sequencing, that is, each gene in a tumor can only obtain one gene mutation information or gene expression information. Intra‐tumor heterogeneity (ITH) refers to the variation of subclonal structures driven by the cancer genome.^[^ ^281^ ^]^ The ITH reflects the number of distinct clones that make up the tumor and the degree of their genetic diversity, and their combination influences tumor invasiveness. As antineoplastic drugs destroy only a subset of cancer cells, ITH exists to promote the evolution of the remaining tumor subregions, leading to immune escape and resistance to treatment.^[^ ^282^ ^]^ Therefore, ITH is emerging as a highly acceptable genomic marker with important implications for the prediction of treatment efficacy. For example, a pan‐cancer analysis showed that patients with low ITH had better survival and response to antitumor therapy,^[^ ^283^ ^]^ and the same results can be seen in NSCLC,^[^ ^284^ ^]^ melanoma,^[^ ^285^, ^286^ ^]^ breast cancer,^[^ ^287^, ^288^ ^]^ ccRCC^[^ ^289^ ^]^ and ovarian cancer.^[^ ^290^ ^]^ Currently, biological assays of ITH mostly rely on biopsy of tumor tissue for whole genome sequencing or whole exome sequencing to obtain mutant‐allele tumor heterogeneity through downstream analysis.^[^ ^291^ ^]^ With advances in single‐cell technologies, ITH can be determined by single‐cell genomics, for example, there may be different clonal subpopulations of tumor cells in a tumor, and this subpopulation may change in course of treatment.^[^ ^292^ ^]^ ScRNA‐seq can also depict ITH from another perspective. Cancer cells may be affected by nongenetic factors (such as metabolic environment, hypoxia, and immune infiltration) to produce huge differences at the transcriptional level, which may affect treatment decisions. For example, when the tumor is found to be in the state of immune microenvironment activation through scRNA‐seq, immunotherapy may be a better choice, otherwise, other treatment modalities (such as chemotherapy or targeted therapy) should be used.^[^ ^293^, ^294^ ^]^

### 6.2 Impact of ITH on Radiogenomics Research

Tissue samples obtained by needle biopsy often represent only a small part of the tumor area,^[^ ^295^ ^]^ and it is unreasonable to represent the biological characteristics of the whole tumor tissue. Biopsy in different regions of the same glioma revealed spatial heterogeneity of EGFR amplification or IDH mutations, which can be inferred from imaging by probability distribution algorithm.^[^ ^192^, ^296^, ^297^ ^]^ Similar problems have also been found in prostate cancer.^[^ ^172^ ^]^ To solve this problem, we need to perform spatial registration of puncture area and imaging ROI and statistically use “sample” as the basic unit, rather than “patient”. For example, Udayakumar et al. constructed a radiogenomic approach to identify different molecular subtypes of lesions through spatially matched RCC tissue‐imaging samples, on the basis of which treatment decisions could lead to better treatment outcomes.^[^ ^158^ ^]^ A prominent issue arising from this is that most imaging studies only use a single tumor (typically the primary tumor or the largest tumor) for segmentation,^[^ ^298^ ^]^ and as the subject of research to predict clinical outcomes or genomic profiles. However, studies have reported that there are large molecular biological differences between primary tumors and metastatic lesions of tumors, which are reflected in gene mutation,^[^ ^299^ ^]^ immune cell phenotype,^[^ ^300^, ^301^ ^]^ and metabolic patterns.^[^ ^302^, ^303^ ^]^ Metastases directly affect the clinical outcomes of patients, so it is also necessary for radiomics studies to include metastases from tumors in the analysis when considering ITH to improve model efficacy. There is not only spatial heterogeneity but also temporal heterogeneity. Temporal heterogeneity refers to the dynamic evolution of the tumor genome during the disease process.^[^ ^304^ ^]^ This means the need for multiple biopsies, a higher economic burden, and a greater likelihood of complications. Imaging provides a unique advantage in that it can comprehensively evaluate the whole tumor characteristics and conduct longitudinal monitoring in the follow‐up imaging of daily clinical work. Although imaging often provides a macro‐scale assessment, imaging features are in fact pathophysiology driven and therefore comprehensive by imaging approaches to the tumor, longitudinal assessment to obtain potential pathophysiology information can be very valuable,^[^ ^305^ ^]^ using imaging data from baseline and subsequent follow‐up, for which delta imaging can be performed, it has been reported to be more accurate in predicting the long‐term prognosis of patients.^[^ ^306^, ^307^ ^]^

### 6.3 Habitat Imaging is a New Radiomic Method

Current imaging‐based tumor heterogeneity assessments tend to use histograms, or texture analysis^[^ ^308^ ^]^ to assess voxel‐to‐voxel heterogeneity and complexity.^[^ ^309^ ^]^ However, although these methods measure the degree of heterogeneity and spatial complexity, grouping of similar voxels is not possible because spatial information on voxels is discarded. In addition, traditional radiomic methods are usually performed on the basis of the whole tumor, which presupposes that tumor heterogeneity is evenly distributed throughout the tumor, ignoring local phenotypic differences within the tumor.^[^ ^310^ ^]^

Unlike traditional methods, a new approach can divide tumors into subregions containing voxel clusters with similar characteristics, often referred to as “habitats”, based on the multidimensional characteristics of individual voxels in an unsupervised clustering manner.^[^ ^311^, ^312^ ^]^ Habitat imaging brings new ideas to the assessment of ITH. Habitat imaging is also known as “sub‐regional imaging” or “Habitat analysis” because it involves a similar description of the ecological environment as in ecology. It involves a personalized description of each voxel within and around the tumor region and uses clustering methods (such as K‐means, Affinity Propagation, and Agglomerative Clustering) to group similar voxels together. Considering that subregions composed of voxels with similar imaging features share similar biological properties and tumor microenvironments, thus, habitat imaging can better present and quantify ITH.^[^ ^313^, ^314^ ^]^ In addition, habitat analysis can provide real spatial information on tumor heterogeneity. Several previous studies have confirmed the association of tumor habitats with pathological mechanisms and found that certain habitat subregions are highly correlated with treatment resistance, which could explain their poor treatment response and prognosis.^[^ ^315^ ^]^ Considering the noninvasive and reproducible nature of imaging, habitat imaging can not only assess tumor invasiveness and predict treatment efficacy, but also assess regional changes in tumor lesions during treatment, to reduce the evaluation error caused by ITH.

From the point of view of analogy, radiomics develops from semantic features, whole ROI features, to habitat imaging techniques for individual voxel. Biological sequencing methods have evolved from polymerase chain reaction‐based single gene test, bulk sequencing, and to then single‐cell or spatial sequencing. From 2009 to 2014, many studies have analyzed the relationship between simple semantic features and the expression of single or countable genes. From 2015 to date, most studies on the relationship between the radiomics from whole ROI and bulk sequencing have been conducted. It can be predicted that in the future, with the increasing popularity of habitat analysis algorithms and the cost reduction of single‐cell or spatial sequencing techniques, the interaction analysis of habitat characteristics, gene mutation or expression of individual cells, and spatial location can enhance our multi‐dimensional understanding of ITH.

### 6.4 The Current Imaging Assessment of ITH and its Biological Background

There is no recognized method for evaluating ITH in imaging. At present, some preliminary studies have been carried out focusing on the radiomic features and their biological interpretation. Aerts et al. defined a group of radiomic features related to tumor heterogeneity and found that two imaging heterogeneity features (“Grey Level Nonuniformity” and “Grey Level Nonuniformity HLH”) were closely related to the prognosis of tumor patients, and these features were related to the cell cycling pathways characterized by the transcription group, which may represent the increase in the proliferative capacity of tumors.^[^ ^115^ ^]^ Zwirner et al. subsequently found that radiomics‐derived tumor heterogeneity was associated with FAT1 mutations in head and neck cancer using the above two features as well as the “run length non uniformity feature”.^[^ ^250^ ^]^ Su et al. used the heterogeneity‐related features in the first order and texture features to build the heterogeneity index (HI) based on the similarity network fusion algorithm. The results showed that the HI of breast cancer was not only directly related to the heterogeneity of genomics or pathomics, but also could effectively stratify the survival of patients.^[^ ^316^ ^]^ Through multi‐omics analysis, it was found that HI was associated with cell proliferation, cell adhesion, and metabolic reprogramming. It was also found that although HI could suggest the increase of tumor mutational burden, it did not cause an effective anti‐tumor immune response. Recently, Li et al. designed a new heterogeneity score, ITHscore, which can measure the distribution changes of clustering patterns in radiomics. They found that ITHscore was related to epithelial‐mesenchymal transition activation and could stratify the prognosis of patients.^[^ ^317^ ^]^ In summary, the present preliminary studies suggest that image‐derived heterogeneity scores are mainly related to tumor proliferation, metabolism, hypoxia, and epithelial‐mesenchymal transition. Based on this, it is not difficult to speculate that tumors with high heterogeneity tend to have more aggressive malignant biological behaviors, resulting in shortened survival of cancer patients.

## 7 Prediction of Intratumoral Immune Microenvironment by Radiogenomics

### 7.1 The Efficacy of Immunotherapy is Related to the Immune Microenvironment

Immunotherapy relies on patients’ own immune systems to recognize and kill cancer cells,^[^ ^318^ ^]^ avoiding the side effects of conventional radiation or chemotherapy and significantly improving survival and quality of life.^[^ ^319^ ^]^ Immunotherapy, represented by three monoclonal antibodies that block programmed death protein 1, programmed death protein 1 ligand, and cytotoxic T‐lymphocyte antigen‐4, has been widely used.^[^ ^320^ ^]^ However, the objective response rate of immunotherapy is low, only around 20% in most tumors.^[^ ^321^, ^322^ ^]^ There are still great challenges in the treatment of most solid tumors. It is suggested that we need reliable biological markers to select patients who may benefit from immune checkpoint inhibitors in order to avoid unnecessary economic burdens and potential complications.^[^ ^323^ ^]^ The tumor immune microenvironment is considered to be a determinant of tumor biology and an important regulator of antitumor drugs.^[^ ^324^ ^]^ There are two major immune cell lineages within the immune microenvironment, which are diverse populations of lymphocytes and myeloid cells.^[^ ^325^ ^]^ Lymphocytes mainly include helper T cells, regulatory T cells, cytotoxic T cells (CD8 T cells), natural killer cells, and B lymphocytes;^[^ ^326^ ^]^ Myeloid cells are a class of cells with considerable heterogeneity within tumors, including dendritic cells, monocyte, macrophage granulocytes, and myeloid‐derived suppressor cells.^[^ ^327^ ^]^ Characterization of immune cells helps to assess the potential for anti‐tumor immune responses and provides selection information for immunotherapy patients.^[^ ^328^ ^]^ RNA‐seq is often used in radiogenomics to obtain the abundance of infiltration of various types of immune cells within a tumor. The rationale for its calculation can be MCP, xCell, and ESTIMATE,^[^ ^329^ ^]^ which use GSEA as a framework, and it can also be a deconvolution‐based tool including CIBERSORT, EPIC, DeconRNASeq, or TIMER.^[^ ^330^ ^]^ Notably, a range of NGS data based on RNA‐seq can be used to extrapolate the type and abundance of immune cells crudely, but further development of higher‐resolution tools or reduction in the cost of single‐cell sequencing is required to meet the needs of large‐scale sequencing of the current large cohort.^[^ ^331^ ^]^ Although previous studies have analyzed the correlation between imaging and immune‐related genes,^[^ ^80^, ^332^ ^]^ models need to be established on this basis to create the possibility of clinical transformation.

### 7.2 Lymphocytes

Based on four cohorts, Sun et al. developed a radiomic model for predicting CD8 T cell numbers, which has been shown to be associated with quantification of tumor infiltration in pathology in a variety of solid tumors,^[^ ^333^ ^]^ and it can stratify the survival of patients receiving immunotherapy. Subsequently, in a study with six independent clinical trials, this radiomic model has also been shown to stratify the prognosis of cancer patients who receive immunotherapy in combination with radiotherapy.^[^ ^334^ ^]^ Zheng et al. constructed models to predict intratumoral CD8A expression using the MRI features of bladder cancer, helping to predict patient prognosis and immunotherapy sensitivity.^[^ ^335^ ^]^ The radiomic model can also predict T cell abundance within head and neck squamous cell carcinoma or HCC tumors^[^ ^336^, ^337^, ^338^ ^]^ or CD3 mRNA expression within gliomas.^[^ ^339^ ^]^ Using a radiogenomic model to accurately quantify infiltrating natural killer cells within NSCLC, Meng et al. found that predicted natural killer cell abundance could accurately stratify patient survival.^[^ ^340^ ^]^ The study purpose of workflow VB is clear, that is to use lymphocyte abundance as the model output for model training, but the study using workflow BI has also explored a considerable number of lymphocyte‐related prediction models. For example, the biological basis of models constructed to predict tumor responses may include interferon immune responses, T cell activation, antigen processing, and presentation.^[^ ^241^, ^341^, ^342^ ^]^ When predicting the long‐term survival or recurrence of patients, T cell and lymphocyte activation and positive regulation of T cell mediated cytotoxicity are one of the important biological bases of the prediction model.^[^ ^67^, ^84^, ^138^, ^168^, ^343^ ^]^

### 7.3 Myeloid Cells

Various myeloid cells regulate anti‐tumor immune capacity in different ways, with M1 macrophages playing a role in promoting anti‐tumor immunity and M2 macrophages promoting tumor progression and suppressing the tumor immune microenvironment.^[^ ^344^ ^]^ Using MRI radiomics, Chen et al. developed a prediction model called RIB that allows non‐invasive assessment of the immune microenvironment of high grade gliomas, particularly, the abundance of M2 macrophages.^[^ ^64^ ^]^ Similarly, Kim et al. used multimodal MRI imaging to also predict the immunophenotype of high grade gliomas, with an area under the curve (AUC) of 0.798 for M2 macrophages.^[^ ^345^ ^]^ Li et al. constructed a predictive model for patient survival in glioma by workflow BI, using bulk RNA‐seq as well as single‐cell transcriptome sequencing for biological interpretation, and it was found that the model score was correlated with the infiltration of macrophages in the tumor.^[^ ^346^ ^]^ Similar results were obtained in gastric cancer, where the biological background of the predictive model for progression‐free survival was predominantly macrophage‐related.^[^ ^100^ ^]^

### 7.4 Non‐Invasive Detection of Immunotherapy Markers or Immue‐Related Subtypes

Non‐invasive detection of clinically available immunotherapy markers by workflow CA or VB is a common research model. To date, three main biomarkers are commonly used in the field of immunotherapy: microsatellite instability, tumor mutational burden, and expression levels of programmed death protein 1 ligand in tumor tissues.^[^ ^347^, ^348^ ^]^ Non‐invasive detection of programmed death protein 1 ligand can be performed by either a deep‐learning approach^[^ ^349^, ^350^, ^351^ ^]^ or conventional radiomic approaches,^[^ ^342^, ^352^, ^353^ ^]^ similarly, tumor mutational burden or microsatellite instability can also be classified using deep learning methods^[^ ^354^, ^355^ ^]^ or conventional radiomic methods.^[^ ^356^, ^357^, ^358^, ^359^, ^360^, ^361^, ^362^, ^363^, ^364^, ^365^ ^]^ Imaging‐immune subtypes of tumors are currently under development. Sun et al. constructed a noninvasive predictive model using CT images from 2600 patients with gastric cancer in nine cohorts, to predict the abundance of lymphocyte and myeloid cells in gastric cancer, and to group imaging‐immune subtypes accordingly, which can effectively predict the efficacy of immunotherapy for gastric cancer.^[^ ^366^ ^]^ In addition, several immune subtypes of tumors have been constructed based on genomics or gene expression profiling that can guide the response to immunotherapy,^[^ ^367^, ^368^ ^]^ thus radiologists will use workflow VB to perform noninvasive detection of immune subtypes, and preliminary studies have already been conducted in gliomas,^[^ ^369^, ^370^ ^]^ head and neck squamous cell carcinoma,^[^ ^371^ ^]^ and ccRCC.^[^ ^372^ ^]^

## 8 PET Transmits Information Beyond Metabolism Through Radiogenomics

### 8.1 High FDG Uptake is Associated with Activation of Multiple Cancer‐Related Pathways

For the first time, Gevaert et al. found a significant correlation between NSCLC image features from PET/CT and metagenesis using paired gene expression microarrays, and metagenesis can be predicted by image features with an accuracy of 59–83%.^[^ ^114^ ^]^ NF‐κb is a key molecule that commonly activates signaling pathways within tumors and regulates glucose metabolism.^[^ ^373^, ^374^, ^375^ ^]^ Based on this, Nair et al. found a positive correlation between FDG uptake and NF‐κb expression in lung cancer.^[^ ^376^, ^377^ ^]^ NF‐κb is also an essential condition for epithelial‐mesenchymal transition activation. Yamamoto et al. subsequently found that higher SUVmax in the NSCLC represented higher transcription levels of TGF‐β, Matrix Metallopeptidase‐9, and versican genes in tumors, which represents the activation of epithelial‐mesenchymal transition and indicates that tumors are more aggressive and resistant to chemotherapy.^[^ ^378^ ^]^ Other studies have also supported this conclusion.^[^ ^379^, ^380^, ^381^ ^]^ In addition, using FDG uptake‐related gene set, the upregulation of FDG found in HCC may also be associated with activation of the mTOR pathway.^[^ ^72^ ^]^ Thus, from an overall perspective, higher FDG uptake tends to represent tumors with a more malignant phenotype,^[^ ^382^, ^383^ ^]^ which may be associated with the activation of multiple intratumoral signaling pathways.

### 8.2 PET and Gene Mutations of Tumor

Correlation analysis between PET information and gene mutations is a commonly used research model, and the correlation between EGFR mutation and PET information is the most extensively studied. Results from preclinical studies indicate that EGFR mutations or KAS mutations cause activation of the EGFR/KRAS/MEK/ERK signaling pathway,^[^ ^384^, ^385^, ^386^ ^]^ which subsequently regulates the expression of C‐MYC and increases the expression levels of GLUT1 and HK2 genes.^[^ ^387^ ^]^ This may eventually lead to an increase in the glycolytic flux of tumor cells. However, studies analyzing PET information from 100 patients with NSCLC have found that low SUV values may be associated with EGFR mutations,^[^ ^388^ ^]^ and several studies have reported similar results.^[^ ^389^, ^390^ ^]^ Conversely, other studies have reported negative or contradictory results.^[^ ^391^, ^392^, ^393^, ^394^ ^]^ Therefore, a larger cohort is required to determine this relationship. Since the mutation status of EGFR may not be determined by PET parameters, other researchers have turned to radiomics or deep learning. Wei et al. analyzed PET or CT images using a 2D small residual convolutional network model, which can predict the EGFR mutation status of NSCLC (AUC = 0.86) and guide patients in making treatment decisions.^[^ ^110^ ^]^ Similar studies also believe that the combination of PET radiomics can significantly improve the prediction ability relative to conventional PET parameters.^[^ ^395^ ^]^ Similar to EGFR, the relationship between KRAS mutation and conventional PET parameters has also produced conflicting results,^[^ ^131^, ^396^ ^]^ but radiomics using PET can also predict KRAS mutation.^[^ ^131^, ^397^ ^]^ Kong et al. used PET radiomics to predict O_6_ methylguanine‐DNA‐methyltransferase promoter methylation in glioma but also found that the conventional PET parameters were not related to O_6_ methylguanine‐DNA‐methyltransferase promoter methylation.^[^ ^200^ ^]^

### 8.3 Feasibility of Using PET to Predict Immune Microenvironment

In earlier studies, some researchers reported that FDG‐PET parameters could predict the treatment response of patients to immune checkpoint inhibitors,^[^ ^398^, ^399^ ^]^ and some studies also reported negative results.^[^ ^400^, ^401^ ^]^ Due to the metabolic competition between tumor cells and immune cells,^[^ ^402^ ^]^ studying the relationship between FDG uptake and tumor immune microenvironment is helpful in explaining the clinical findings. Li et al. found that the density of CD8 T cells was negatively correlated with FDG uptake,^[^ ^403^ ^]^ but the opposite results were obtained in other studies.^[^ ^404^, ^405^, ^406^ ^]^ During immunotherapy, the changes of FDG may also be related to Treg cells. Leveraging the profound relationship between metabolism and immunity, Park et al. developed and validated a biomarker based on deep learning^[^ ^407^ ^]^ to predict the CytAct score (average of granzyme A and perforin 1 expression) obtained from RNA‐seq. The biomarker generated from PET can noninvasively evaluate the immune microenvironment of lung adenocarcinoma and the efficacy of immune checkpoint inhibitors. Similarly, PET imaging was also used to predict the expression of CD8A.^[^ ^408^ ^]^

## 9 Looking for the New Significance of Radiomics in Multi‐Omics Study

### 9.1 The Classic Paradigm of Multi‐Omics Study

Machine learning is the core technology of multi‐omics studies. According to the two methods of machine learning, we can divide multi‐omics studies into unsupervised learning and supervised learning.^[^ ^409^ ^]^ Supervised learning is often used for prediction when given an input label or an output label, such as clinical outcomes in patients with tumors (workflows VB and MP are typical supervised learning). Unsupervised multi‐omics data analysis generally infers new data clusters from any unlabeled input data, without any prior knowledge, to analyze and learn about the underlying patterns and connections between data.^[^ ^410^ ^]^ Multi‐omics analysis through unsupervised learning can often uncover novel subtypes of tumors to facilitate better patient stratification. For example, in the non‐negative matrix factorization, the mRNA and microRNA information of ovarian cancer are projected into a common coordinate system, and a multidimensional module is formed according to the weight of the projection direction, to find new molecular types of ovarian cancer.^[^ ^411^ ^]^ An extension of principal component analysis, Joint and Individual Variation Explained, decomposes groups of learned data inputs into joint structures, individual structures, and residual noise across data types, and based on this, glioblastoma multiforme can be characterized in a completely new way.^[^ ^412^ ^]^ Using iCluster analysis, a novel molecular subtype associated with the prognosis of sarcoma patients was defined after the integration of DNA copy number, DNA methylation, and mRNA and miRNA expression data.^[^ ^413^ ^]^ It is also possible to take advantage of complementarities in multi‐omics data, and to compute and fuse Similarity networks in multi‐omics data, an approach known as similarity network fusion, which is reported to be useful when similarity network fusion utilizes multi‐omics data to identify cancer subtypes, its performance is much better than that of a single omics approach or integrated approaches.^[^ ^414^ ^]^ In summary, multi‐omics study is the frontier field in oncology research at present. Using the complementary information of multi‐omics data to mine potential available molecular targets or molecular subtypes will promote the development of basic medical research and aid in clinical decision‐making. Radiomics can also provide another dimension of tumor information for multi‐omics studies. However, at present, it is mainly used as a substitute or supplementary data for the main omics data.^[^ ^415^ ^]^

### 9.2 The Current Role of Radiomics in Multi‐Omics Study

**Figure 4:**
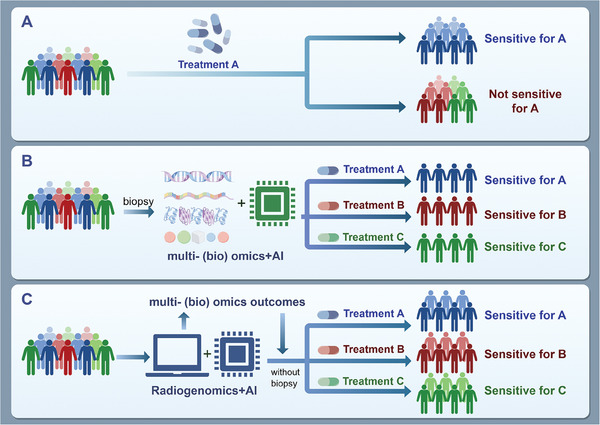
Achieving non‐invasive precision medicine with radiogenomics. A) Conventional treatment. B) Treatment based on multi‐omics data to achieve precision medicine. C) Treatment based on multi‐omics data to achieve non‐invasive precision medicine. Created with Figdraw.com (ID: UOOAUa42ca).

In fact, in most radiogenomics studies, radiomics, as well as at least one other omics type, are often involved, whereas in radiogenomics‐related workflows 1–5, none of the workflows used this research workflow in 9.1 (juxtaposing radiomics with other omics and simultaneously substituting into the multi‐omics research model to generate relevant tumor subtypes), this may be due to two reasons: 1) genomics, transcriptomics, and proteomics are subject to the Central Dogma that a gene is represented in different dimensions, and there are potential biological correlations between different kinds of omics, but there is no corresponding dimension in the Central Dogma for radiomics, a similar problem has arisen in metabolomics (the storage format of metabolites is not at the gene level, making it difficult for machine learning to integrate metabolomics and other omics).^[^ ^416^ ^]^ 2) radiomics tends to provide a comprehensive characterization of the tumor (such as 3D tumor segmentation), whereas, for biological sequencing, only a part of the tumor tissue is needed to obtain data; The presence of ITH reduces the feasibility of juxtaposing radiomics with other omics (ITH have been discussed in section 5.2). In summary, this research model still awaits more powerful multi‐omics integration algorithms to look for potential associations of radiomics with other omics. From the perspective of clinical accessibility, it is difficult to carry out high‐cost multi‐omics sequencing for every cancer patient in daily clinical work, but it is a work with strong practical significance to predict the subtype by imaging with workflow VB (**Figure** **4**). The supervised learning mode represented by workflow MP is widely used in the study of radiomics + other omics, where they tend to use radiomics in conjunction with other omics to jointly predict clinical outcomes,^[^ ^142^, ^247^, ^248^, ^417^ ^]^ but this workflow can get caught up in the “curse of dimensionality” of data, where the efficiency and accuracy of many statistical methods decrease dramatically,^[^ ^418^ ^]^ therefore, efficient feature selection methods and classifiers that can process high‐dimensional data are needed.

## 10 Conclusions, Challenges Faced by Clinical Transformation and Future Trends

### 10.1 More External Validation is Required

Although a considerable number of radiogenomics prediction models have been published so far, these models were validated in the external validation or independent validation of the study itself. We urgently need researchers to verify the previously published prediction models using publicly available codes. Such an approach not only enhances the credibility and reliability of the models but also facilitates the comparison and optimization among them. For example, Sun et al. externally re‐validated the radiomic prediction model of intratumoral CD8 cells developed two years ago and obtained a high prediction accuracy.^[^ ^333^, ^334^ ^]^ Predicting EGFR mutations is a common mode of study in radiogenomics studies, and Kohan et al. validated this in this center cohort using the EGFR prediction model developed by Tu et al., with the AUC decreasing from 0.78 to 0.69.^[^ ^419^, ^420^ ^]^ Similarly, Kim et al. externally re‐validated a predictive model of O_6_ methylguanine‐DNA‐methyltransferase promoter methylation in glioblastoma multiforme from the first‐place Brats 2021 challenge, but they yielded negative results,^[^ ^201^, ^421^ ^]^ which reminds us of the need for greater caution and comprehensiveness in the process of model selection and validation. Therefore, among the many published prediction models, it is necessary to select a prediction model with strong generalization to achieve clinical transformation. In addition, radiogenomic studies involving transcriptome sequencing can utilize Workflow GV for supplementary validation on larger public databases.

### 10.2 Clinically Accessible Prediction Models

In addition to potential problems with predictive accuracy, another issue hindering clinical transformation is that most radiogenomics requires manual segmentation of the tumor ROI, which takes longer if the ROI is multilayered. This is difficult to meet the clinical needs, thus emphasizing the urgent need for deep learning and automatic/semi‐automatic segmentation software that can accurately identify tumor lesions in clinical settings. At the same time, the traditional machine learning model is usually composed of multiple independent modules, each module is an independent task, and its result will affect the next task, thus affecting the whole training model.^[^ ^422^ ^]^ The end‐to‐end model uses a joint model to learn parameters from multiple models, and all intermediate operation processes are included in the neural network, which is no longer divided into multiple modules.^[^ ^423^ ^]^ Compared with the non‐end‐to‐end conventional machine learning model, the learning of the end‐to‐end model omits the image annotation work required before each learning task.^[^ ^424^ ^]^ Tumor ROI annotation is usually tedious and error‐prone. Although various companies have developed a large number of automatic or semi‐automatic software, manual verification is still necessary. In addition, considering that traditional radiogenomics models usually need to go through the feature extraction and modeling stages, different parameter adjustment processes will lead to different results, and the end‐to‐end model avoids the tedious feature extraction process, thus improving the prediction efficiency and prediction robustness of the model. Previously, Uhm et al. proposed an end‐to‐end deep learning model for the differential diagnosis of five major histological subtypes of renal tumors by multiphase CT, which achieved good predictive efficacy (AUC = 0.889) and was validated in the TCIA cohort (AUC = 0.855).^[^ ^425^ ^]^ Compared with radiologists, this end‐to‐end model can achieve similar or better diagnostic performance in identifying a wide range of renal tumor stages. Similarly, Wang et al. developed an end‐to‐end deep learning prediction model, which only needs means to frame the region where the tumor is located, without fine segmentation of the tumor edge, to obtain the EGFR mutation prediction probability of the tumor.^[^ ^426^ ^]^ Therefore, end‐to‐end learning greatly improves image segmentation accuracy, computational efficiency, and reproducibility in radiogenomics through direct image information transformation of input and output.^[^ ^427^ ^]^ It is expected to be widely used in medical work in the future to solve current clinical problems. Another notable area is the rapid development of large language models, represented by ChatGPT, Claude, and Gemini, which have made significant progress in recent years. In the medical field, they have demonstrated powerful capabilities, not only efficiently extracting information from clinical data but also accurately handling information extraction and error correction tasks for imaging reports.^[^ ^428^, ^429^ ^]^ Particularly important is that current large language models have acquired image recognition capabilities and shown great potential in diagnosing pathological images in multiple studies.^[^ ^430^, ^431^, ^432^ ^]^ Even more striking is that these models' performance in diagnosing medical images is considered comparable to that of radiologists, all without any pre‐training or image segmentation steps, aligning with actual clinical needs.^[^ ^433^ ^]^ Today, large language model technology stands at the forefront of the AI field, indicating its potential to become indispensable tools in radiogenomic research in the future.

### 10.3 Noninvasive or Low‐Cost Omics Combination

As stated above, the “gene” part of radiogenomics represents the microscopic level of tumors. This microscopic information often relies on the acquisition of samples through needle biopsy or surgical resection, which needs to consider the economic cost, potential complications, and patient tolerance. Currently, typical representatives of high‐cost omics technologies are single‐cell transcriptomics and spatial transcriptomics.^[^ ^434^ ^]^ These advanced sequencing methods can provide extremely precise analysis of cellular components and detailed revelation of gene expression patterns for each cell type.^[^ ^435^ ^]^ However, most studies currently focusing on single‐cell sequencing face the challenge of limited sample size and this limitation makes it particularly difficult to construct high‐precision models using workflow MP. The main reasons lie in the scarcity of sample size and the high complexity of data dimensions within individual samples. Specifically, each sample contains an extremely large number of data dimensions, and existing machine learning techniques still struggle when dealing with such small sample sizes and high‐dimensional data, making it difficult to extract effective and accurate information, thereby constraining the improvement of model performance.^[^ ^436^, ^437^ ^]^ Therefore, we need to focus on non‐invasive and low‐cost omics. This includes three types of omics, namely, liquid biopsy, pathomics, and radiomics. Tumors release a variety of components into the circulatory system, including tumor cells, DNA, RNA, and proteins.^[^ ^438^, ^439^ ^]^ The circulating‐tumor DNA can be comprehensively analyzed by sequencing, which may be able to project the characteristics of the primary tumor without biopsy. At present, preliminary studies have found that imaging information is related to circulating‐tumor DNA and cell‐free DNA.^[^ ^440^ ^]^ In addition, combining imaging information with SNPs of blood genomics can also improve the efficiency of prediction models.^[^ ^69^ ^]^ Pathomics can perform in‐depth analysis based on existing pathological images.^[^ ^441^ ^]^ For example, it can provide information about lymphocyte infiltration and genome in tumors using artificial intelligence technology.^[^ ^442^, ^443^, ^444^ ^]^ Therefore, the combination mode of noninvasive or low‐cost omics around liquid biopsy, pathomics and radiomics is an important breakthrough for noninvasive precision therapy in the future, and also an opportunity for the development of radiogenomics.

### 10.4 Construction of Multi‐Omics Database Containing Imaging Information

TCGA has played an important role in the field of radiogenomics and promoted progress in this field. We can assume that the number of radiogenomics databases worldwide will be rapidly increased by retrospectively supplementing imaging data in the current multi‐omics database that lacks imaging data. It includes not only bulk sequencing databases, but also a large number of single‐cell sequencing or spatial transcriptome databases. Encouragingly, CPTAC, APOLLO‐5, and CMB databases are currently under construction. They will present scientists with a new pan‐cancer genome‐imaging cohorts in the future, which will significantly accelerate the research progress and clinical translation of radiogenomics, and derive a series of multi‐omics algorithms. We expect that these goals will eventually be achieved as sequencing costs fall and artificial intelligence technologies evolve. This process will stimulate more opportunities for interdisciplinary collaboration, promote deep integration across multiple fields such as biology, medicine, and computer science, and jointly drive the leapfrog development of radiogenomics and even the entire field of precision medicine.

### 10.5 Large‐Scale Prospective Clinical Trials Focusing on Radiogenomics Models are Necessary

So far, although the number of radiomics publications has increased year by year, only a few studies have actually carried out prospective research, and even fewer have been transformed into clinically available tools. According to the clinical trial registry (clinicaltrials.gov), nearly 30 radiogenomics studies are currently being conducted or have already completed the recruitment of clinical trials (**Table** **2**), which mainly focus on cancer research, including glioma, colorectal cancer, lung cancer, esophageal cancer, head and neck cancer, cholangiocarcinoma, prostate cancer, bladder cancer, endometrial cancer, ovarian cancer, pancreatic cancer, breast cancer, cervical cancer and other cancers, indicating that more and more researchers have carried out prospective cohort collection in radiogenomics. Nevertheless, compared with more than 300 radiogenomics‐related studies published in the past ten years, less than one‐tenth of the studies that have been registered and are carrying out clinical trials. Retrospective cohort studies, while providing abundant data resources for radiogenomics, are limited by inherent issues such as missing data and selection bias, which restrict the accuracy and reliability of research findings.^[^ ^445^, ^446^ ^]^ In contrast, prospective cohort studies, through the continuous and systematic inclusion of participants who meet specific research and model requirements, not only enhance the internal validity of studies but also effectively reduce bias, providing a more solid foundation for validating the reliability and robustness of models.^[^ ^447^, ^448^ ^]^ Therefore, to accelerate the translation of radiogenomic research findings into clinical practice, there is an urgent need for more prospective cohort studies to validate and optimize existing models and methods. Simultaneously, strengthening interdisciplinary collaboration across medicine, biology, and computer science, as well as establishing more comprehensive data sharing and collaboration mechanisms, are crucial for driving the sustained development of the radiogenomics field.

**Table 2:** Registration of Tumor‐related Radiogenomics in the ClinicalTrials.gov.

Tumor type	Interventions	Status	Row^a)^
Glioma	Diagnostic Test: Assess the response glioma to radiochemotherapy using radiogenomics‐based AI model	Recruiting	1
Glioblastoma	Other: Observational only	Not yet recruiting	11
Astroblastoma		Recruiting	22
CNS Sarcoma		Recruiting	22
Ependymoma of Brain		Recruiting	18
Pediatric Brain Tumor	Diagnostic Test: ^11^C‐MET‐PET/RMN	Recruiting	19
Colon Cancer Stage II/ III		Recruiting	2
Rectal Cancer		Recruiting	14
Lung Cancer		Not yet recruiting	3
Cancer, Lung		Completed	4
Non‐Small Cell Lung Cancer	Diagnostic Test: Radiomic signature	Completed	12
NSCLC, PET/CT, Biopsy	Diagnostic Test: ^18^F‐FDG PET/CT and PET/CT‐guide targeted biopsy in another group of participants	Unknown	16
Lung Cancer		Enrolling by invitation	25
Cancer of Esophagus		Completed	4
Cancer of Head and Neck		Completed	4
Gallbladder Cancer	Diagnostic Test: CT scan	Recruiting	5
Prostate Cancer Adenocarcinoma	Radiation: Hypo‐FLAME study	Completed	20
Bladder Cancer Stage II	Diagnostic Test: MRI imaging of the pelvis/bladder Diagnostic Test: genomic analysis of tumor	Terminated	7
Endometrial Cancer	Other: transcriptomic profiling	Recruiting	8
Endometrial Cancer	Genetic: gene expression analysis Genetic: gene rearrangement analysis Genetic: polymorphism analysis	Unknown	17
Ovarian Cancer		Recruiting	9
Ovarian Cancer	Diagnostic Test: Germinal BRCA	Recruiting	10
Pancreatic Cancer	Other: Blood Sample collection Other: Tissue sample collection Other: Data collection	Recruiting	13
Pancreatic Adenocarcinoma	Drug: FOLFIRINOX Device: Electroporation	Withdrawn	23
Pancreatic Adenocarcinoma	Device: Electroporation Drug: gemcitabine Drug: nab‐paclitaxel	Completed	24
Breast Cancer	Genetic: gene expression analysis Genetic: gene rearrangement analysis Genetic: polymorphism analysis	Unknown	17
Breast Cancer		Unknown	21
Cervical Cancer	Genetic: gene expression analysis Genetic: gene rearrangement analysis Genetic: polymorphism analysis	Unknown	17
Pediatric Solid Tumor		Recruiting	18
BCOR ITD Sarcoma		Recruiting	22
Unclassified Tumor, Malignant		Recruiting	22

^a)^ Reference from ClinicalTrials.gov

## Conflict of Interest

The authors declare no conflict of interest.

## Author Contributions

Y.G., T.L., and B.G. contributed equally to this work. Y.G. designed the manuscript; Y.G., T.L., and B.G. drafted the manuscript; B.G. and T.L. made contributions to the part of radiomics; Y.H. made contributions to the part of AI; S.W. made contributions to the part of high‐throughput technologies. L.Y. and C.Z. revised the manuscript. All authors involved in manuscript writing and final approval of the manuscript.

## Supporting information



Supporting Information
